# Trained immunity in diabetes and hyperlipidemia: Emerging opportunities to target cardiovascular complications and design new therapies

**DOI:** 10.1096/fj.202301078R

**Published:** 2023-10-01

**Authors:** Katherine A. Robinson, Naveed Akbar, Kajus Baidžajevas, Robin P. Choudhury

**Affiliations:** ^1^ Division of Cardiovascular Medicine, Radcliffe Department of Medicine University of Oxford Oxford UK

**Keywords:** cardiovascular, diabetes, epigenetics, macrophages, metabolism, trained immunity

## Abstract

Some metabolic diseases, such as diabetes and hyperlipidemia, are associated with a state of inflammation, which adversely affects cardiovascular health. Emerging evidence suggests that long‐term hyperactivation of innate immune cells and their bone marrow progenitors, termed trained immunity, functions to accelerate atherosclerosis and its complications in cardiometabolic diseases. This review will focus on how trained immunity is established, particularly through metabolic and epigenetic reprogramming, to cause persistent and deleterious changes in immune cell function, even after the original stimulus has been corrected or removed. Understanding the mechanisms driving maladaptive trained immunity and its fundamental contribution to cardiovascular disease might enable the development of novel disease‐modifying therapeutics for the reduction in cardiovascular risk in diabetes, hyperlipidemia, and related cardiometabolic states.

## Graphical abstract

Innate immune cell activation during metabolic diseases (e.g., diabetes, obesity, and hyperlipidemia) leads to metabolic and epigenetic reprogramming of cells (such as macrophages and their bone marrow progenitors) that causes persistent and deleterious changes in cell function, termed trained immunity or innate immune memory. The presence of trained immunity in metabolic diseases leads to accelerated cardiovascular diseases (e.g., atherosclerosis).

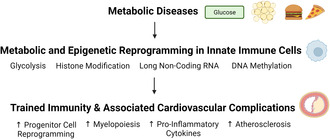

**Abbreviations**
acetyl‐CoA: acetyl coenzyme A
ASCVD: atherosclerotic cardiovascular disease
ATMs: adipose tissue macrophages
BCG: Bacillus Calmette–Guérin
CRP: high‐sensitivity C‐reactive protein
DAMPs: damage‐associated molecular patterns
FH: fumarate hydratase
H3K27ac: histone 3 lysine 27 acetylation
H3K4me1: histone 3 lysine 4 monomethylation
H3K4me3: histone 3 lysine 4 trimethylation
HIF1α: hypoxia‐inducible factor 1α
HITI: hyperglycemia‐induced trained immunity
IL‐1β: interleukin 1 beta
IL‐6: interleukin 6
Ldlr: low‐density lipoprotein receptor
Lp(a): lipoprotein(a)
LPS: lipopolysaccharide
LXRs: liver X receptors
mTOR: mechanistic target of rapamycin
NK: natural killer
oxLDL: oxidized LDL
OxPLs: oxidized phospholipids
PAMPs: pathogen‐associated molecular patterns
PBMCs: peripheral blood mononuclear cells
PRRs: pattern recognition receptors
SAT: subcutaneous adipose tissue
TCA: tricarboxylic acid cycle
TIH: transient intermittent hyperglycemia
TLR: toll‐like receptor
TNF‐α: tumor necrosis factor alpha
VAT: visceral adipose tissue
WD: western diet

## 1 INTRODUCTION

Innate immune cells have usually been considered to respond to stimuli de novo, without the ability to form an immunological memory. However, it was found that even organisms that lack adaptive immune responses are protected against reinfection with pathogens.^1^ The phenomenon of “trained immunity,” first described in 2011, is an ability of innate immune cells to acquire a persistent, non‐specific immunological memory of prior functionally important exposures, which results in an enhanced or modified response to subsequent immune stimuli.^2^ This concept was demonstrated in humans in 2012,^3^ when Bacillus Calmette–Guérin (BCG) vaccination in healthy volunteers was shown to lead to the functional reprogramming of monocytes, such that they elicited an enhanced pro‐inflammatory response to secondary challenge with unrelated pathogens. This modified response persisted for at least 3 months after vaccination.^3^ In studies of severe combined immunodeficient mice, which lack functional T and B lymphocytes and thus adaptive immune responses, BCG vaccination reduced mortality caused by a secondary, potentially lethal infection.^3^

Initially, trained immunity was discovered in circulating monocytes (i.e., peripheral trained immunity), but the long‐term persistence of trained immunity is reliant upon reprogramming at the level of bone marrow progenitor cells that replenish short‐lived peripheral cells (i.e., central trained immunity).^4^ In addition to circulating monocytes and their bone marrow progenitor cells, a trained phenotype has been reported in other innate immune cell populations, including dendritic cells,^5^ natural killer (NK) cells,^6^ and neutrophils.^7^, ^8^, ^9^ Furthermore, single‐cell RNA sequencing has revealed a large heterogeneous response of trained immunity signature cytokines and chemokines among trained human monocyte/macrophage subpopulations following lipopolysaccharide (LPS) restimulation.^10^ Specifically, unsupervised clustering analysis of trained immunity phenotypes in macrophages revealed three distinct subpopulations of equal proportions: (i) macrophages with enhanced expression of genes encoding chemokines and pro‐inflammatory cytokines; (ii) macrophages with enhanced expression of chemokines only; and (iii) non‐trained cells, which are cells with low trained immunity phenotypes.^10^ Thus, it will be important to further elucidate the functional consequences of these subpopulations.

Trained immunity develops after the binding of pathogen‐associated molecular patterns (PAMPs), such as β‐glucan and BCG, to cells of the innate immune system via pattern recognition receptors (PRRs) expressed on their cell surface and cytoplasm. This leads to a long‐term metabolic and epigenetic reprogramming, with consequent enhanced secretion of pro‐inflammatory cytokines (e.g., tumor necrosis factor alpha [TNF‐α], interleukin 6 [IL‐6], and IL‐1β) and chemokines (e.g., C‐X‐C motif chemokine ligand [CXCL]‐9–11) upon secondary stimulation. In this context, trained immunity provides an important defense against microbial infections; however, it can also be induced by certain endogenous factors, such as hyperglycemia,^11^, ^12^ hyperlipidemia,^13^ consumption of Western diet,^14^ obesity,^15^, ^16^ oxidized LDL (oxLDL),^17^, ^18^ and heme.^19^ The development of this maladaptive trained immunity, in response to sterile inflammatory triggers, contributes to inflammation‐related pathology such as can occur in cardiovascular complications of metabolic diseases.

In this review, we describe the cellular and molecular mechanisms of trained immunity, specifically metabolic and epigenetic changes fundamental to the long‐term hyperinflammatory reprogramming of innate immune cells. We then discuss accumulating evidence that implicates maladaptive trained immunity as a novel mechanism driving inflammation, which can yield important insights for the treatment and prevention of cardiovascular complications of metabolic diseases.

## 2 METABOLIC AND EPIGENETIC REPROGRAMMING

**FIGURE 1:**
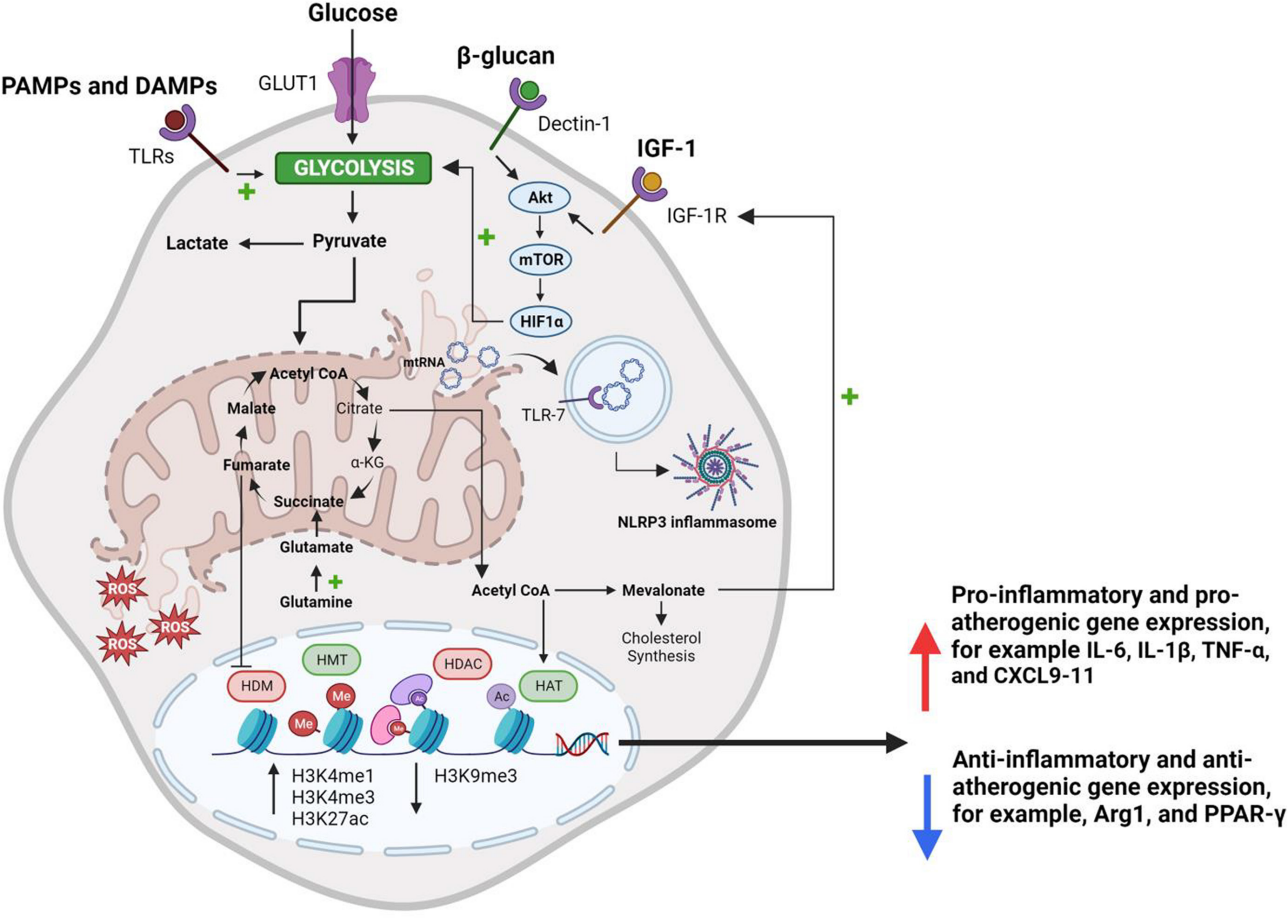
Cardiometabolic disease alters cellular metabolism and drives epigenetic reprogramming to induce increased pro‐inflammatory and pro‐atherogenic gene expression. Illustration of the long‐term reprogramming of metabolic and epigenetic pathways in macrophages, characteristic of deleterious trained immunity, that occurs in cardiometabolic diseases. In macrophages, increased levels of pathogen‐associated molecular patterns (PAMPs) or damage‐associated molecular patterns (DAMPs) bind to pattern recognition receptors, such as toll‐like receptors (TLRs), and cause metabolic reprogramming leading to increased glycolysis regulated by the mechanistic target of rapamycin (mTOR) hypoxia‐inducible factor 1α (HIF1α) pathway. HIF1α leads to the induction of key glycolytic genes, and this shift in central glucose metabolism leads to an increase in pyruvate, which is converted into acetyl coenzyme A (acetyl‐CoA) and citrate and then exported to the cytosol to be converted back into acetyl‐CoA by ATP citrate lyase. Excess cytosolic acetyl‐CoA enters the mevalonate pathway leading to the accumulation of the metabolite mevalonate, which activates insulin growth factor‐1 receptor (IGF‐1R) signaling and enhances HIF1α activation and glycolytic flux. In addition, the upregulation of glutaminolysis (conversion of glutamine to glutamate) induces the accumulation of tricarboxylic acid (TCA) cycle metabolites such as succinate, fumarate, and malate during training. Changes in metabolite levels (i.e., acetyl CoA and fumarate) induce changes in gene expression and cell function via the stabilization of HIF1α and regulation of epigenetic enzymes. By influencing the regulation of epigenetic enzymes such as histone methyltransferases (HMT), histone demethylases (HDM), histone acetyltransferases (HAT), and histone deacetylases (HDAC), PAMPs and DAMPs can induce long‐term reprogramming through deposition of key histone modifications including histone 3 lysine 4 mono‐ and tri‐methylation (H3K4me1 and H3K4me3) and histone 3 lysine 27 acetylation (H3K27ac) and removal of histone 3 lysine 9 trimethylation (H3K9me3) at the promoters or enhancers of pro‐inflammatory genes. Finally, the accumulation of fumarate (via fumarate hydratase suppression) might also induce mitochondrial stress and immunostimulatory mitochondrial RNA (mtRNA) and reactive oxygen species (ROS) release, activating immunological RNA sensors such as toll‐like receptor 7 (TLR‐7). This results in NLRP3 inflammasome activation and consequent pro‐inflammatory cytokine release. α‐KG indicates α‐ketoglutarate; Akt, protein kinase B; GLUT‐1, glucose transporter‐1; IGF‐1, insulin growth factor‐1; IL‐6, interleukin‐6; IL‐1β, interleukin‐1β; TNF‐α, tumor necrosis factor‐α; CXCL9‐11, chemokine (C‐X‐C motif) ligand 9‐11; Arg1, arginase 1; PPAR‐γ, peroxisome proliferator‐activated receptor gamma; + indicates enhancement; bold text indicates upregulation. Figure created with BioRender.com.

The induction of trained immunity by various PAMPs and damage‐associated molecular patterns (DAMPs) is dependent upon the long‐term reprogramming of metabolic and epigenetic pathways in innate immune cells, which enables an enhanced inflammatory response upon restimulation (Figure 1). At a molecular level, immune stimulation results in the convergence of multiple regulatory pathways, including changes in cellular metabolism (e.g., glycolysis, the tricarboxylic acid [TCA] cycle, and oxidative phosphorylation) and the related induction of genome‐wide epigenetic reprogramming (e.g., changes in chromatin organization and accessibility, transcription of long non‐coding RNAs [lncRNAs], and DNA methylation), essential to the formation of functional trained immunity programs in innate immune cells and their progenitors. Indeed, it is well established that changes in cellular metabolites from glycolysis and the TCA cycle (e.g., acetyl coenzyme A [acetyl‐CoA] and fumarate) act as substrates and cofactors for chromatin‐modifying enzymes, including histone methyltransferases and demethylases, and histone acetyltransferases and deacetylases, leading to specific changes in histone methylation and acetylation in relation to genes involved in innate immune responses.

### 2.1 Metabolic reprogramming

Perhaps it is not surprising that “immunity” and the homeostatic functions of “immune cells” are intertwined with metabolic function since they have evolved with a backdrop of cellular “metabolism” in which substrate status is fundamental to cellular functions. Crucially, trained immunity is associated with a metabolic shift of central glucose metabolism from oxidative phosphorylation to aerobic glycolysis (the “Warburg effect”), regulated via the mechanistic target of rapamycin (mTOR) hypoxia‐inducible factor 1α (HIF1α) pathway.^20^ This metabolic reprogramming results in increased glucose consumption and higher lactate production. Many trained immunity pathways converge on glycolysis and, importantly, the inhibition of glycolysis abolishes the trained immunity phenotype in some models,^12^, ^17^, ^20^, ^21^ demonstrating the significance of glycolysis as a key metabolic pathway in the formation of innate immune cell memory. In line with this, associations between single nucleotide polymorphisms (SNPs) in key glycolytic genes, including 6‐phosphofructo‐2‐kinase/fructose‐2,6‐biphosphatase 3 (*PFKFB3*) and phosphofructokinase (*PFKP*), have been found to influence the training capacity of monocytes isolated from healthy volunteers.^17^

Although we and others have focused on the role of glycolysis, there are simultaneous and important changes in other metabolic pathways and metabolites that contribute to the development of trained immunity. For example, glutaminolysis (the conversion of glutamine into glutamate) also plays a central role in the induction of trained immunity. Specifically, increased rates of glutaminolysis replenish TCA cycle intermediates, resulting in the accumulation of succinate, fumarate, and malate.^22^ The central role of fumarate has been exemplified by its ability to dose‐dependently induce training in monocytes in vitro.^22^ Mechanistically, fumarate accumulation contributes to the trained immunity phenotype in at least two ways: (i) by inhibiting KDM5 histone lysine demethylases and thus influencing monocyte epigenetic reprogramming^22^ and (ii) through inhibition of HIF1α proteasomal degradation, which maintains the increased glycolytic flux necessary for trained immunity.^23^ It must be noted that succinate accumulation has also been found to stabilize HIF1α and prevent its proteasomal degradation, leading to an upregulation of glycolytic enzymes and enhanced pro‐inflammatory IL‐1β production during inflammation.^24^ However, unlike fumarate, succinate was not able to induce trained immunity in monocytes in vitro.^22^ Moreover, Hooftman et al.^25^ identify increased argininosuccinate synthase (*Ass1*) expression and concomitant decreased fumarate hydratase (encoded by *Fh1* in mice and *FH* in humans) expression, which led to cytosolic fumarate accumulation, fumarate‐mediated protein succination, suppressed IL‐10, and increased TNF‐α secretion following exposure of bone marrow‐derived macrophages (BMDMs) and human peripheral blood mononuclear cells (PBMCs) to acute LPS stimulation. Interestingly, FH suppression was found to induce mitochondrial stress and immunostimulatory mitochondrial RNA (mtRNA) release, activating immunological RNA sensors such as toll‐like receptor 7 (TLR‐7).^25^ Indeed, FH suppression has been detected in whole blood cells from patients with systemic lupus erythematosus^26^ and monocytes isolated from patients with type 1 diabetes^11^ that exhibit a trained immunity phenotype, highlighting a potential pathogenic role for FH suppression, mitochondrial stress, and concomitant mtRNA release during trained immunity.

In addition to the role of fumarate, excess acetyl‐CoA (generated via a truncated TCA cycle where pyruvate is converted into acetyl‐CoA and citrate and then exported to the cytosol to be converted back into acetyl‐CoA by ATP citrate lyase) enters the mevalonate pathway. This leads to accumulation of the metabolite mevalonate, which dose dependently induces trained immunity in vitro via the activation of insulin growth factor‐1 receptor (IGF‐1R)‐mTOR signaling.^21^ This creates a positive‐feedback loop whereby glycolysis promotes mevalonate accumulation, which subsequently amplifies glycolysis via IGF‐1R signaling. In support of the role of mevalonate, monocytes from patients with a mevalonate kinase deficiency, termed hyperimmunoglobulin D syndrome (HIDS), accumulate mevalonate and have a constitutive trained immunity phenotype at both the immunological and epigenetic levels.^21^

An “opposite” of trained immunity is innate immune tolerance or immunoparesis, wherein the cell is less able to activate inflammatory gene transcription upon pro‐inflammatory restimulation.^27^ Immunomodulatory metabolite, itaconate, derived from cis‐aconitate by the enzyme cis‐aconitate decarboxylase (ACOD1), encoded by immunoresponsive gene 1 (*IRG1*), triggers antioxidant and anti‐inflammatory responses through TCA cycle remodeling and is a central component of the inhibitory effects during immune tolerance.^28^ Interestingly, β‐glucan‐induced trained immunity counteracted tolerance via inhibition of *IRG1* expression, and consequently itaconate production, in a model of human endotoxemia.^28^ Thus, the itaconate synthesis pathway represents an important regulatory node between tolerance and trained immunity and could be harnessed therapeutically in inflammation‐related diseases associated with trained immunity.

### 2.2 Epigenetic reprogramming

Epigenetic reprogramming is essential to the establishment and maintenance of long‐term innate immune memory. Indeed, training can be abolished following the inhibition of epigenetic enzymes with methylthioadenosine (MTA, a non‐selective methyltransferase inhibitor) and ITF2357 (ITF, a histone deacetylase inhibitor).^11^, ^18^, ^20^, ^29^, ^30^ Two of the most well‐studied epigenetic modifications enhanced in trained immunity include histone 3 lysine 4 trimethylation (H3K4me3) at promoters, and histone 3 lysine 27 acetylation (H3K27ac) at both promoters and distal enhancers of genes involved in immunity / inflammation and metabolism, leading to their increased transcriptional activation in monocytes and macrophages.^17^, ^18^, ^20^, ^21^, ^22^, ^29^, ^30^, ^31^, ^32^ The acquisition of H3K4 monomethylation (H3K4me1) at distal enhancers has also been observed in trained immunity.^31^, ^33^ For example, macrophage pro‐inflammatory stimulation resulted in the unveiling of latent enhancers, marked by deposition of H3K4me1. Once unveiled, many of these enhancers did not return to a latent state when stimulation ceased; instead, they persisted and mediated a faster, stronger, and non‐specific response to restimulation in a manner dependent on H3K4me1, which may reflect a direct reading of this mark by histone acetyltransferases.^33^ The unveiling, and the subsequent retention, of a repertoire of latent enhancers can be considered an epigenomic memory of prior stimulation. Interestingly, Saeed et al.^31^ identified persistent H3K4me1 at decommissioned enhancers in trained cells, which supports a hypothesis that trained immunity employs latent enhancers as a mechanism for establishing an epigenomic memory of prior stimulation. The role of repressive histone modifications in trained immunity has been explored to a lesser extent; however, repressive histone modification H3K9me3 was found to be decreased at promoters of pro‐inflammatory genes during β‐glucan training, contributing to their increased transcriptional activation.^30^

In addition, given the relationship between metabolism and epigenetics and their importance in trained immunity, there is potential to uncover novel metabolism‐associated histone modifications that have not previously been considered, such as histone lactylation. Indeed, histone lactylation acts as an endogenous “lactate clock” that directly stimulates gene transcription in the late phase of M1 macrophage polarization to induce the expression of M2‐like homeostatic genes involved in wound healing and repair, including arginase 1 (*Arg1*).^34^ It is plausible that this mechanism is defunct in trained macrophages that display resistance to induction of anti‐inflammatory processes that mediate regression or repair. The relevance of histone lactylation to cardiovascular disease is confirmed by its ability to regulate early homeostatic gene expression (including leucine‐rich alpha‐2‐glycoprotein 1 (*Lrg1*), vascular endothelial growth factor A (*Vegf‐a*), and interleukin‐10 (*Il‐10*)) in the bone marrow and peripheral monocytes promoting cardiac repair post‐myocardial infarction.^35^

Furthermore, as described above, the specific activation of epigenetic enzymes has also been observed in trained immunity phenotypes. Inhibition of H3K4 methyltransferase Set7 (*SETD7*) during training in vitro attenuated pro‐inflammatory cytokine production following restimulation with LPS, and *Setd7* knock‐out (KO) mice are unable to mount an enhanced response to secondary challenge in vivo, highlighting the importance of Set7 for trained immunity.^32^ The authors described a role for Set7 in the accumulation of TCA cycle metabolites associated with trained immunity via the Set7‐dependent H3K4me1 enrichment at distal enhancers of TCA cycle enzymes malate dehydrogenase 2 (*MDH2*) and succinate dehydrogenase B (*SDHB*; conversion of succinate to fumarate).^32^ In addition, the expression of histone methyltransferase G9a, also known as Euchromatic histone lysine methyltransferase 2 (EHMT2), which mediates H3K9 methylation, was decreased following the induction of trained immunity.^36^ Pharmacological inhibition of EHMT2 in monocytes amplified trained immunity responses, as shown by increased pro‐inflammatory cytokine production and increased metabolic rate.^36^ Moorlag et al.^37^ identified a strong association between KDM4 histone demethylases, which promote gene transcription by removing the repressive histone modification H3K9me3, and trained immunity responses. Confirming this, inhibition of KDM4 proteins in vitro by the small‐molecule JIB‐04 significantly decreased trained immunity responses induced by either β‐glucan or BCG due to increased levels of the repressive modification H3K9me3 at genes important for the induction of glycolysis. Moreover, the increase in H3K27ac in β‐glucan‐trained monocytes has been linked to reduced NAD^+^‐dependent class III histone deacetylase, Sirtuin‐1, expression.^20^

In addition to changes in histone modifications, there is strong evidence that immune gene‐priming lncRNAs (IPLs) are central to the establishment of trained immunity. Training mediated by β‐glucan epigenetically reprogrammes immune genes by upregulating IPLs.^38^ The insertion of IPL, upstream master LncRNA of the inflammatory chemokine locus (UMLILO), into the chemokine topologically associating domain in mouse macrophages resulted in training of chemokine genes.^38^

## 3 TRAINED IMMUNITY IN CARDIOMETABOLIC DISEASES

Trained immunity has evolved under continuous evolutionary pressure to provide enhanced protection against invading pathogens^1^; however, the development of maladaptive trained immunity in response to sterile inflammatory triggers contributes to inflammation‐related disease pathology. It is well established that activation of inflammatory processes adversely affects cardiovascular health in the context of metabolic diseases, most prominently type 1 and type 2 diabetes, hypercholesterolemia, and obesity. Sterile inflammatory triggers present in these metabolic diseases (e.g., hyperglycemia, oxLDL, lipoprotein(a) (Lp(a)), and pro‐inflammatory cytokines) have been shown to induce trained immunity, resulting in chronic low‐grade inflammation and accelerated atherosclerotic cardiovascular disease (ASCVD). The potential significance of trained immunity in ASCVD is highlighted by its presence in patients with established symptomatic atherosclerosis.^39^

Further to this, epidemiological data have revealed that mothers exposed to chronic inflammation during pregnancy can predispose offspring to an increased risk of developing cardiovascular and metabolic (e.g., obesity and diabetes) disease in childhood and early adulthood.^40^ This is true for pre‐gestational diabetes, including type 1 and type 2 diabetes, gestational diabetes, and maternal obesity.^41^, ^42^, ^43^ Indeed, there is evidence that trained immunity induced by microorganisms is transmitted across generations in both vertebrates^44^ and invertebrates,^45^, ^46^ which could have important clinical implications for the link between maternal metabolic disease and cardiovascular health in offspring. However, it must be noted that the intergenerational transmission of trained immunity could not be validated in a second study in vertebrates.^47^ There is evidence that systemic intrauterine fetal programming displays hallmarks of trained immunity, in particular epigenetic changes, which are critical in the transmission of cardiometabolic disease from mothers to their offspring (reviewed in^48^). For example, a recent study found that DNA methylation biomarkers in peripheral blood cells maintained across the first year of life could discriminate children born to mothers who suffered from obesity or gestational diabetes, and crucially, these alterations affected genes related to immune system activation (*ETS1, ITGB2*, and *TNF* family), metabolism (*FN3K, SLC38A4, SLC35F3*, and *CPT1B*), and epigenetics (*HDAC4, PRDM16*).^49^ Maternal obesity and diabetes could constitute serious risk factors in relation to the appearance of cardiometabolic diseases in the offspring and it remains to be studied whether parental trained immunity is transmitted across generations to predispose offspring to cardiometabolic diseases. If proven, the identification and treatment of trained immunity in cardiometabolic diseases in women of childbearing age could help mitigate the acquisition of trained immunity and its associated risk of cardiovascular disease in the next generation.

### 3.1 Diabetes

Diabetes causes extensive morbidity and mortality through vascular complications such as ASCVD and acute myocardial infarction. Treatments to prevent the vascular complications of diabetes have largely focused on lowering blood glucose; however, these do not result in a commensurate reduction in cardiovascular risk.^50^, ^51^, ^52^ This persistent risk of cardiovascular complications, even after glucose‐lowering therapy, has been termed “metabolic memory,” and may be linked to the development of long‐term hyperglycemia‐induced trained immunity or “HITI”. In support of this, studies in diabetes suggest that such innate immune cell reprogramming can last months, even years.^53^

Specifically, hyperglycemia promotes progression of ASCVD through remote effects on the bone marrow.^54^ For example, diabetic mice have increased numbers of circulating neutrophils and Ly6C^hi^ monocytes, reflecting a hyperglycemia‐induced proliferation and expansion of bone marrow myeloid progenitors with associated release of monocytes into the circulation.^55^ Further to this, glucose variations or transient intermittent hyperglycemia (TIH) was found to promote myelopoiesis more intensely leading to the persistent recruitment of inflammatory monocytes into atherosclerotic lesions and accelerating pre‐clinical atherosclerosis.^56^ Systemic hyperglycemia induced increased glucose uptake via glucose transporter, GLUT1, in neutrophils to promote the production of neutrophil‐derived alarmins, S100A8/S100A9, which drive myelopoiesis via interaction with the glucose‐inducible receptor for advanced glycation end products (RAGE) on common myeloid progenitor cells.^56^ Myeloid‐restricted deletion of *Slc2a1* (GLUT1) or pharmacological inhibition of S100A8/S100A9 reduced TIH‐induced myelopoiesis and atherosclerosis. Indeed, plasma S100A8/S100A9 levels correlate with leukocyte counts and coronary artery disease in patients with type 1 diabetes,^55^ suggesting that targeting the S100A8/S100A9‐RAGE axis could reduce atherosclerosis progression and cardiovascular events in diabetic individuals with adequate glycemic control.

In addition to driving increased myeloid cell production, hyperglycemia alters the function of bone marrow cells and their macrophage progeny through mechanisms that depend on both metabolic and epigenetic reprogramming. For example, high extracellular glucose results in a shift towards glycolysis and accumulation of TCA cycle intermediates with consequent enhanced pro‐inflammatory and pro‐atherogenic gene expression and decreased anti‐inflammatory gene expression in mouse macrophages.^12^ Crucially, these changes persist even after the cells are subsequently restored to a normal physiological glucose environment. Inhibition of glycolysis, using dichloroacetate and 2‐DG, inhibited changes in inflammatory gene expression and pro‐atherogenic functions.^12^ Furthermore, hyperglycemia induced persistent changes in chromatin accessibility and histone methylation and acetylation (i.e., increased H3K4me3 and H3K27ac) at relevant genomic loci including key genes in inflammation (*Il‐6*) and glucose metabolism (hexokinase 1; *Hk1*), which led to increased transcriptional activation in diabetic hematopoietic stem cells (HSCs) and differentiated macrophages.^12^ Transcription factor runt‐related transcription factor 1 (RUNX1), which has previously been associated with immunological memory, was implicated in the transcriptional activation of key trained immunity genes, and pharmacological inhibition of RUNX1 removed these manifestations of training in vitro. The functional significance of HITI in the development of atherosclerosis was recently demonstrated through transplantation of bone marrow obtained from diabetic mice (induced by streptozotocin) into atherosclerosis‐prone, normoglycemic low‐density lipoprotein^−/−^ (Ldlr^−/−^) recipient mice.^12^ Significantly, the bone marrow from diabetic mice retained its hyperglycemic memory and markedly accelerated atherosclerosis development in recipient mice confirming a cardiovascular disease‐relevant and persistent form of trained immunity. In addition, recipient mice also showed an increase in plaque macrophage content and lipid‐rich necrotic core, which accelerates disease progression and impairs atherosclerosis lesion resolution in vivo.^12^ Equivalent findings of HITI were confirmed in atherosclerotic plaque macrophages and circulating PBMCs from patients with type 2 diabetes, consistent with a potential role in human disease.^12^

Further studies investigate the nature of epigenetic reprogramming in peripheral monocytes exposed to hyperglycemic conditions. For example, THP‐1 monocytes cultured in conditions of high extracellular glucose (25 mM) led to increases in H3K4me2 and H3K9me2 at promoter regions of genes associated with chronic inflammation, including *IL‐1A* and *IL‐8*, which was confirmed in peripheral monocytes isolated from patients with type 1 and type 2 diabetes.^57^ Moreover, high‐glucose culture of monocytes, mimicking diabetic conditions, can lead to the recruitment of the key p65 subunit of nuclear factor kappa‐light‐chain enhancer of activated B cells (NF‐κB) and histone acetyltransferases to the promoters of inflammatory genes such as TNF‐α and cyclooxygenase 2 (COX‐2), resulting in histone acetylation (e.g., H3K9ac, H3K14ac, H4K5ac, H4K8ac, and H4K12ac), chromatin remodeling, and increased transcriptional activation.^58^ These changes were confirmed in monocytes isolated from patients with type 1 and type 2 diabetes. Notably, overexpression of histone deacetylase isoforms inhibited the p65‐mediated upregulation of TNF‐α transcription.^58^ Additional studies of hyperglycemia‐induced training of monocytes from patients with type 1 diabetes demonstrated that epigenetic reprogramming was dependent upon upregulation of the mixed lineage leukemia (MLL) lysine methyltransferases, which methylate H3K4.^11^ Treatment with the MLL inhibitor, menin‐MLL, during the process of trained immunity repressed the pro‐inflammatory phenotype. Other significantly upregulated methyltransferases include *SETD1A* and *SETD1B*; however, these were not further explored.^11^

Clearly, there is a potentially important role for HITI in the perpetuation of chronic inflammation and acceleration of ASCVD in patients with diabetes (and consequent inhibition of processes that mediate ASCVD regression or repair) even after glucose‐lowering therapy, which might explain why targeting elevated glucose, without also targeting processes of inflammation, is ineffective in reducing cardiovascular risk.

### 3.2 Hypercholesterolemia

Multiple studies have implicated hypercholesterolemia and oxLDL as sterile inducers of trained immunity, which might explain some of the “residual risk” of cardiovascular disease despite the use of cholesterol‐lowering statin therapy. The possibility was emphasized by a recent analysis^59^ of >30 000 patients (>76% type 2 diabetes) from three trials. In patients in whom LDL cholesterol had been lowered to contemporary standards, residual cardiovascular risk was associated not with LDL cholesterol, but with inflammation. Elevated high‐sensitivity C‐reactive protein (CRP) (highest quartile vs. lowest) conferred increased cardiovascular and all‐cause mortality (2·68‐fold *p* < ·0001 and 2·42‐fold *p* < ·0001, respectively). CRP levels are driven by IL‐6, a cardinally activated gene in HITI.^12^

Bekkering et al.^13^ found that monocytes from patients with familial hypercholesterolemia are characterized by a trained immune phenotype. Importantly, lowering cholesterol levels using a 3‐month statin treatment did not reverse monocyte hyperresponsiveness in this patient population, with key epigenetic marks including increased H3K4me3 and decreased H3K9me3 at the promoters of pro‐inflammatory cytokines (e.g., TNF‐α) remaining unaffected by statin treatment.^13^ As with hyperglycemia, the effects of hypercholesterolemia‐induced reprogramming were detectable at the level of the bone marrow progenitor cells. For example, bone marrow aspirates from patients with familial hypercholesterolemia taken before and 3 months after cholesterol‐lowering statin therapy showed increased gene expression in pathways involved in hematopoietic migration and myelomonocytic skewing.^60^ Interestingly, statin therapy reversed myelomonocytic skewing but the transcriptomic reprogramming of monocyte‐associated inflammatory and migratory pathways persisted, indicating the presence of centrally trained immunity.^60^

Further verification of the effects of hypercholesterolemia on bone marrow progenitor cells and its clinical relevance to ASCVD has been evidenced in pre‐clinical models. For example, the activation of hematopoietic progenitor cell proliferation and skewed development towards myeloid lineages especially granulocytes and inflammatory monocytes persisted following bone marrow transplantation from hypercholesterolemic Ldlr^−/−^ mice into normocholesterolemic recipients, which resulted in increased leukocyte migration into the artery, with consequent significant increased plaque size with a more advanced phenotype.^61^ Another study demonstrated hypercholesterolemia‐associated monocytosis, with the production of Ly6C^hi^ monocytes that adhered to activated endothelium, infiltrated lesions, and became lesional macrophages with impaired Ly6C^hi^ to Ly6C^lo^ conversion.^62^ These data confirm the cardiovascular disease‐relevant nature of hypercholesterolemia‐induced training and provide a further rational basis for the use of anti‐inflammatory drugs to lower residual cardiovascular risk in patients with hypercholesterolemia in addition to statin therapy.

Furthermore, oxLDL resulting from the oxidation of LDL cholesterol can directly activate macrophages to induce a prolonged pro‐inflammatory and pro‐atherogenic phenotype which is sustained by metabolic and epigenetic reprogramming in human monocytes.^18^ Keating et al.^17^ identified that oxLDL‐induced training is critically dependent on intracellular metabolic alterations, including a concomitant upregulation of glycolysis and oxidative phosphorylation as indicated by increased oxygen consumption rate. In support of this, oxLDL‐induced trained immunity was found to induce transcriptional activation of genes enriched in mitochondrial metabolic pathways, and metabolome analysis revealed mitochondrial TCA cycle as the most upregulated pathway, in addition to increases in other mitochondria‐linked pathways such as alanine/aspartate metabolism.^63^ As described above, aspartate metabolism is involved in the LPS‐induced inflammatory activation of macrophages via the inflammatory aspartate–argininosuccinate shunt, which resulted in FH suppression and increased pro‐inflammatory cytokine release, mitochondrial stress, and immunostimulatory mitochondrial nucleic acid release.^25^ Indeed, oxLDL‐induced trained immunity is associated with changes in mitochondrial size, mass, and membrane polarization.^63^ The importance of the mitochondria in supporting cytokine hyperresponsiveness in trained immunity was confirmed using pharmacological inhibitors targeting mitochondrial function, which dose dependently inhibited the production of TNF‐α.^63^ Collectively, these findings recognize the need for further elucidation of the role of the mitochondria in the training of macrophages and their response to sterile inflammatory ligands. Notably, decreasing mitochondrial stress in macrophages can prevent inflammation in ASCVD and decrease atherosclerosis burden in mice,^64^ thus representing a potential therapeutic avenue to treat trained immunity in cardiometabolic disease.

Furthermore, it has previously been shown that liver X receptors (LXRs) are key regulators of cholesterol and fatty acid metabolism, have established roles in the regulation of BCG‐induced trained immunity, and can independently induce training in human monocytes^65^ via the activation of HIF1α‐dependent glycolysis.^66^ Notably, LXRα inhibition in human monocytes blocked oxLDL‐induced trained immunity by preventing the deposition of activating histone marks at the promoters of pro‐inflammatory genes such as *IL‐6* and *TNF‐α*.^67^ These data highlight a role for LXRα in the formation of oxLDL‐induced trained immunity. In addition to oxLDL, Lp(a), the major lipoprotein carrier of phosphocholine‐containing oxidized phospholipids (OxPLs) in plasma has also been associated with the induction of trained immunity in monocytes as evidenced by an increased capacity to transmigrate and produce pro‐inflammatory cytokines upon stimulation.^68^ Moreover, in vitro studies found that Lp(a) augments the pro‐inflammatory response in healthy human monocytes, which was markedly attenuated by inactivating OxPL on Lp(a), which is a recognized DAMP.^68^ This is clinically relevant as subjects with elevated Lp(a) have increased arterial inflammation and enhanced PBMC trafficking to the arterial wall, suggesting that trained immunity might provide a novel link among Lp(a), OxPL, and accelerated atherosclerosis in humans.^68^

### 3.3 Diet induced

Western‐type calorically rich diets (WD), containing numerous immunologically active substances (e.g., high glucose, cholesterol, saturated fatty acids, L‐carnitine, and phosphatidylcholine, which are converted to trimethylamine N‐oxide [TMAO]), are proficient at inducing a chronic inflammatory state that is associated with long‐term innate immune cell reprogramming.^69^ Specifically, in atherosclerotic‐prone Ldlr^−/−^ mice, a 4‐week WD induced the transcriptomic and epigenomic reprogramming of myeloid progenitor cells with skewing towards myelopoiesis, which persisted after 4 subsequent weeks of chow diet and resulted in a hyperresponsive phenotype upon restimulation.^14^ Mechanistically, the authors identified the NLRP3 inflammasome and subsequent production of IL‐1β as critical to the WD‐induced trained immunity phenotype. *Nlrp3* deletion in Ldlr^−/−^ mice led to the abolition of WD‐induced systemic inflammation, hematopoiesis, and myeloid precursor reprogramming. Thus, NLRP3 orchestrates WD‐induced training and could be targeted to mitigate the deleterious complications of WD such as cardiovascular disease. The relevance of WD‐induced trained immunity to the development of ASCVD is demonstrated by the significantly increased aortic root plaque size (in the absence of changes in serum cholesterol) following bone marrow transplantation from WD‐fed Ldlr^−/−^ mice into chow‐fed recipients.^70^ Mice reconstituted with WD‐fed bone marrow exhibited hypomethylation of CpG regions in the genes encoding *Pu.1* and interferon regulatory factor 8 (*Irf8*), key transcriptional regulators of monocyte proliferation and macrophage differentiation, and increased numbers of circulating peripheral leukocytes.^70^

### 3.4 Obesity

Obesity is, in part, a chronic inflammatory disorder and is an independent risk factor for cardiovascular disease. Specifically, changes in white adipose tissue (WAT) mass, accompanied by the accumulation of pro‐inflammatory adipose tissue macrophages (ATMs), drive obesity‐associated inflammation and associated cardiovascular disease.^71^ This deleterious phenotypic switch of innate immune cells persists even after obesity‐associated metabolic alterations have been normalized through weight loss, which has been termed an “obesogenic memory”.^15^ Indeed, weight loss increased inflammatory cytokine production to a second activation signal ex vivo in ATMs from previously obese mice.^16^ Further evidence demonstrates that this obesity‐induced trained immunity is exacerbated by complete or partial weight regain (termed weight cycling), which further accelerates cardiometabolic disease.^16^, ^72^

Mechanistically, obesogenic memory is dependent on both metabolic and epigenetic reprogramming. For example, the treatment of healthy mouse macrophages with palmitic acid or adipose tissue conditioned media from obese mice significantly increased maximal glycolysis and oxidative phosphorylation and increased LPS‐induced TNF‐α and IL‐6 production in vitro in a manner dependent on TLR4 signaling.^16^ These effects were impaired by inhibition of mTOR (metformin) or methyltransferase inhibition (MTA), thus confirming this form of innate memory is driven by metabolic and epigenetic changes.^16^ As with other forms of trained immunity, obesity also reprogrammes myeloid cells at the level of the bone marrow,^73^ driving both quantitative increases in myeloid progenitors and the preferential generation of inflammatory ATMs even after serial bone marrow transplantation, an effect that was regulated by hematopoietic MyD88.^73^ As in hyperglycemia‐induced trained immunity, obesity enhanced myelopoiesis in the bone marrow via adipose‐derived production of S100A8/S100A9.^74^ This induced ATM TLR4/MyD88 and NLRP3 inflammasome‐dependent IL‐1β production, which interacted with the IL‐1 receptor on myeloid progenitor cells to stimulate the enhanced production of monocytes and neutrophils.^74^ This exemplifies the existence of a positive feedback mechanism in obesity, whereby the inflamed adipose tissue stimulates the production of more pro‐inflammatory monocytes, which perpetuates further inflammation. Thus, targeting the NLRP3‐IL‐1β signaling axis could reduce adipose tissue inflammation and metabolic disease in obesity. Notably, following influenza infection, the inflamed lung environment promotes the recruitment and retention of inflammatory Ly6C^hi^ monocytes, which are central to the non‐specific protection against secondary infection, whereas the non‐inflammatory lung environment promotes terminal differentiation into steady‐state resident alveolar macrophages.^75^ This is interesting because it highlights that an equivalent reduction in adipose tissue inflammation might aid in the phenotypic switch of recruited monocytes to a steady‐state anti‐inflammatory macrophage population that blunts this positive feedback mechanism in obesity.

Finally, it has been demonstrated in vitro that the distribution of adipose tissue in obese patients can differentially induce trained immunity in peripheral monocytes (Tuijl et al. 2021; doi:10.1093/eurheartj/ehab724.3438). Specifically, both visceral adipose tissue (VAT) and subcutaneous adipose tissue (SAT) from patients with obesity‐induced persistent innate immune cell activation in healthy human monocytes, as assessed by increased cytokine production in response to a secondary stimulus. However, adipose tissue‐secreted metabolites from VAT induced a higher cytokine response when compared to SAT, suggesting that VAT has an enhanced potential to induce trained immunity. Strategies that focus on the reduction in VAT in obese subjects might help to reduce the severity of obesity‐associated trained immunity and its cardiometabolic consequences.

## 4 THERAPEUTIC MODULATION OF TRAINED IMMUNITY IN CARDIOMETABOLIC DISEASES

Cardiometabolic diseases have chronic systemic inflammation underpinned by the persistent and deleterious activation of innate immune cells at the level of the bone marrow and their macrophage progeny. A deeper understanding of the mechanisms driving trained immunity in these diseases should bring opportunities for disease prevention and to develop new disease‐modifying therapies.

### 4.1 Repurposing current pharmaceutical interventions

Clinical trials have demonstrated the importance of decreasing inflammation in the treatment of cardiovascular diseases.^76^, ^77^, ^78^ Significantly, the use of anti‐inflammatory therapeutics targeted against IL‐1β^79^ (CANTOS) leads to a reduction in cardiovascular events independently of lipid lowering in patients with previous myocardial infarction and high CRP. In addition to drugs that target inflammatory cytokines, drugs that inhibit the NLRP3 inflammasome such as colchicine led to a significantly lower risk of cardiovascular events in patients with recent myocardial infarction^80^ (COLCOT) or chronic coronary disease^81^ (LoDoCo2). Thus, highlighting that interventions to mitigate inflammation may also reduce the risk of cardiovascular events in patients with cardiometabolic diseases. There are also other existing drugs that have the potential to interfere with trained immunity. For example, hydroxychloroquine, which has been shown to reduce IL‐6 levels in patients with myocardial infarction^82^ (OXI trial), was found to directly inhibit trained immunity in COVID‐19 patients.^83^ Furthermore, metformin, an activator of AMP kinase and thus inhibitor of mTOR, and widely used drug in the treatment of type 2 diabetes, was found to inhibit trained immunity in humans in vitro and in mice in vivo.^17^, ^20^, ^22^ Notably, monocytes isolated from healthy individuals receiving metformin could not be trained ex vivo with atherosclerosis‐relevant stimulus oxLDL.^17^

### 4.2 Development of new pharmaceutical interventions

Nanomedicine, the application of nanomaterials and nanodevices for the prevention, diagnosis, and treatment of disease, has begun to take a leading role in the setting of cardiovascular disease (extensively reviewed in doi:10.1038/s44161‐023‐00232‐y). Specifically, nanomedicines enable the selective targeting of therapeutics to specific tissues and cell subsets, which improves drug toxicity profiles, enhances drug efficacy, facilitates cellular internalization, and protects against premature metabolism or degradation.

Nanomedicines can efficiently deliver therapeutics (such as small molecules, polymers, RNA therapeutics, and immunoregulatory proteins) to immune cells via direct nanoparticle–phagocyte interactions with myeloid cells and their bone marrow progenitors, and thus hold great promise for the regulation of trained immunity (excellently reviewed in doi: 10.1038/s41578‐021‐00413‐w). Notably, the inhibition of mTOR using myeloid cell‐specific nanomedicines prevented oxLDL‐induced pro‐inflammatory cytokine production in human monocytes in vitro, and rapidly diminished the inflammatory activity of plaque macrophages in vivo, presenting an innovative therapeutic avenue for targeting key signaling pathways in trained immunity for the treatment of atherosclerosis.^84^ Thus, nanomedicines have the potential to develop powerful trained immunity‐regulating therapeutics; however, finding a balance between dampening maladaptive trained immunity to lessen its adverse cardiovascular effects, while maintaining a level of protective trained immunity essential to host defense against infection, will remain a key challenge.

Finally, due to the significant contribution of epigenetic reprogramming to the development and maintenance of the trained immunity phenotype, epigenetic enzymes that write and erase histone modifications are another obvious therapeutic target to prospectively remodel or even reverse the trained immunity‐related cardiovascular complications of metabolic diseases. Indeed, inhibitors of epigenetic enzymes have previously been used to selectively regulate inflammatory responses in human macrophages. For example, inhibitors of the jumonji (JMJ) family of histone lysine demethylases, which demethylate lysine residues in histones in a methylation state and sequence‐specific context, have been used to regulate pro‐inflammatory responses in human macrophages.^85^ Specifically, a small‐molecule catalytic site inhibitor selective for the H3K27me3‐specific demethylase subfamily (KDM6 subfamily members JMJD3 and UTX), called GSK‐J4, reduces LPS‐induced pro‐inflammatory cytokine production by human primary macrophages.^85^ Interestingly, JMJ H3K27me3‐specific demethylases have also been identified as key regulators of cytokine production in human NK cell subsets, which can also adopt a trained phenotype.^86^ Indeed, GSK‐J4 induced a global increase in the repressive promoter‐associated H3K27me3 modification at effector cytokine genes, which resulted in a reduction in interferon gamma (IFN‐γ), TNF‐α, granulocyte–macrophage colony‐stimulating factor (GM‐CSF), and IL‐10 levels in cytokine‐stimulated NK cells.^87^ This profound anti‐inflammatory effect of GSK‐J4 was confirmed in NK cell subsets isolated from peripheral blood or tissue from patients with rheumatoid arthritis, suggesting that histone demethylase inhibition has broad utility for modulating immune and inflammatory responses in a chronic inflammatory disease setting. In addition, GSK‐J4 can repress T helper 17 cell inflammation via a global increase in H3K27me3 levels, which induced profound metabolic reprogramming (e.g., a reduction in mitochondrial biogenesis) and suppression of pro‐inflammatory IL‐17 cytokine levels with concomitant anti‐inflammatory effects.^88^ Thus, inhibitors of epigenetic enzymes may provide a potential therapeutic avenue to remodel deleterious genome‐wide epigenetic reprogramming in inflammatory diseases linked to trained immunity in multiple cell types. However, there are many barriers to the widespread use of epigenetic inhibitors in the treatment of chronic diseases. Certainly, the future application of nanomedicines for the specific delivery of epigenetic drugs to innate immune cells might facilitate their safe and effective use in the treatment and management of inflammatory diseases.

## 5 CONCLUSIONS AND FUTURE PERSPECTIVES

In trained immunity, immunomodulatory factors present in metabolic diseases promote the progression of cardiovascular disease through mechanisms that depend on both the metabolic and epigenetic reprogramming of bone marrow cells and their monocyte/macrophage progeny leading to the transcriptional activation of pro‐inflammatory and pro‐atherogenic genes and ensuing acceleration of atherosclerosis. Crucially, these functional alterations persist even after the original stimulus, such as hyperglycemia or hypercholesterolemia, is corrected or removed.

More sophisticated understanding of the underlying metabolic and epigenetic processes driving these profound and sustained effects on immune cell function should bring opportunities to target cardiovascular disease and its prevention. Specifically, novel insights into the role of emerging metabolites such as fumarate and lactate, metabolic pathways involving mitochondria, and epigenetic modifications such as histone lactylation in the phenotype of trained immunity will enable the development of new disease‐modifying therapeutics to address the clinical challenges related to the management of metabolic diseases, such as diabetes and hyperlipidemia, and their associated mortal cardiovascular complications.

## AUTHOR CONTRIBUTIONS

Robin Choudhury designed and supervised the review, and the revision and final approval of the manuscript; Katherine Robinson undertook the literature review research and manuscript writing. Naveed Akbar and Kajus Baidžajevas further added, revised, and updated the manuscript. All authors have read and approved the final manuscript.

## DISCLOSURES

The authors declare no conflict of interest.

## Data Availability

Data sharing is not applicable to this review as no datasets were generated or analyzed in this review.
